# Corrected QT interval on the electrocardiogram after liver transplantation: Surrogate marker of poor clinical outcomes?

**DOI:** 10.1371/journal.pone.0206463

**Published:** 2018-10-26

**Authors:** Seung-Hwa Lee, Myungsoo Park, Kyoung-min Park, Hye-bin Gwag, Jungchan Park, Jeayoun Kim, Gyu-Seong Choi, Suk-Koo Lee, Gaab Soo Kim

**Affiliations:** 1 Department of Medicine, Heart, Stroke and Vascular Institute, Samsung Medical Center, Sungkyunkwan University School of Medicine, Seoul, Republic of Korea; 2 Department of Medicine, Dongtan Sacred Heart Hospital, Hallym University School of Medicine, Seoul, Republic of Korea; 3 Department of Anesthesiology and Pain Medicine, Samsung Medical Center, Sungkyunkwan University School of Medicine, Seoul, Republic of Korea; 4 Department of Surgery, Samsung Medical Center, Sungkyunkwan University School of Medicine, Seoul, Republic of Korea; University of Toledo, UNITED STATES

## Abstract

**Background:**

Prolongation of corrected QT interval (QTc) on the electrocardiogram is associated with cardiac arrhythmia and sudden death. Changes in the QTc (corrected QT) interval before and after liver transplantation (LT) for the treatment of liver cirrhosis (LC) and its association with clinical outcomes have not been fully evaluated.

**Methods:**

From January 2011 to May 2016, consecutive 516 consecutive recipients were enrolled into LT registry and the median follow-up was 31 months (IQR 12–52). Patients with an available electrocardiogram before LT and 1 month after from LT were analyzed. Patients were divided into 2 groups according to prolonged QTc interval. The patient groups were analyzed separately according whether the electrocardiogram was preoperative or postoperative. The primary outcome was all-cause death during the follow-up period.

**Results:**

A total of 283 patients were enrolled in the study. In the preoperative QTc prolongation group, there was not a significant rate difference in all-cause mortality in multivariate analysis (hazard ratio [HR], 0.94; 95% confidence interval [CI], 0.53–1.66; *P* = 0.26). However, in the postoperative QTc prolongation group, mortality was significantly increased (HR, 1.78; 95%CI, 1.05–3.03; *P* = 0.03) in patients who underwent LT.

**Conclusion:**

In patients who underwent LT for LC, postoperative QTc prolongation on ECG, rather than preoperative, is associated with mortality. Larger clinical trials are needed to support this finding.

## Introduction

Electrocardiogram (ECG) is a record of electrical activity of the heart and routinely performed to detect any cardiac problems. The QT interval in the ECG encompasses ventricular depolarization and repolarization, and shows the processes of myocyte excitation, represented by the action potential.[1–3] Meanwhile, QT interval length varies with heart rate, and the correct interpretation of the QT interval requires correction by heart rate and called corrected QT interval (QTc).[3] QTc prolongation is one marker of cirrhotic cardiomyopathy and is frequently seen in patients with liver cirrhosis (LC) [1–3]. QTc prolongation is also associated with functional re-entry, torsade de pointes, and sudden death in the general population [4,5]. Moreover, LC patients with QTc prolongation show increased mortality [6].

The ultimate treatment for LC is liver transplantation (LT) [7]. Cirrhotic cardiomyopathy could theoretically be reversed by LT, but in some patients, QT prolongation did not change or even worsened [8,9]. LC patients with QT prolongation had increased mortality rates, but QT prolongation itself had no independent effect on mortality after LT, and more than half of patients showed normalized QT intervals after LT [10]. To date, changes in the QT interval after LT and its influence on clinical outcomes have not been fully evaluated. The aim of this study was to investigate the effects of preoperative and postoperative QT prolongation and their association with clinical outcomes in LC patients who underwent LT.

## Methods

### Study population and data collection

The present study was a single-center retrospective study. The study population was enrolled from the LT database of Samsung Medical Center. From January 2011 to May 2016, 516 consecutive LT recipients were selected into the registry. The inclusion criteria were: 1) patients who underwent preoperative ECG and 2D echocardiography, 2) patients with follow-up ECG after 1months of window period, and 3) older than 19 years. The exclusion criteria were: 1) patients who did not undergo preoperative 2D echocardiography and preoperative and postoperative ECG, 2) patients undergoing multiple organ transplantations, and 3) patients without LC. Clinical, laboratory, and outcome data were collected by a trained study coordinator using a standardized case report form and protocol. All participants were analyzed anonymously, and consents were approved by the Institutional Review Board of Samsung Medical Center.

### Definition and outcomes

Diabetes mellitus was defined as a history of type 1 or type 2 diabetes mellitus that was, treated either pharmacologically or through dietary changes. Hypertension was either defined as self-reported use of antihypertensive medications or as systolic blood pressure >140 mm Hg. Resting blood pressure was measured when patients were admitted. Smoking history was defined as an at least 10 pack-year history of tobacco use. Ascites was detected immediately after surgical incision. A preoperative laboratory test, ECG, and 2D echocardiography were conducted as part of the routine preoperative evaluation. The formula for the model for end-stage liver disease (MELD) score was 3.8*log_e_(bilirubin [mg/dL]) + 11.2*log_e_(INR) + 9.6*log_e_(creatinine [mg/dL]) + 6.4*(etiology: 0 if cholestatic or alcoholic, 1 otherwise) [11]. Diastolic dysfunction was defined as in Mitter, et al [12] Left ventricular enlargement was defined as left ventricular end-diastolic diameter over 59mm by M-mode. A postoperative laboratory test was conducted with the follow-up ECG. The primary outcome was all-cause death during follow-up.

### QT prolongation measurement

The ECGs were reviewed, and the QTc (QT corrected for heart rate) interval was calculated by one trained staff member blinded to the patient status. The QT interval was measured from the beginning of the QRS complex to the termination of the T- wave (defined as the return to the isoelectric line) in leads where it was identifiable. The QTc interval was calculated by dividing the QT interval in seconds by the square root of the R-R interval in seconds (Bazett formula) [13]. Prolonged QTc was defined as QTc > 440ms [14]. Patients were divided into 4 groups according to the presence of prolonged QTc preoperatively [QTc prolongation (+), group A; QTc prolongation (-), group B] or postoperatively [QTc prolongation (+), group C; QTc prolongation (-), group D]. Clinical outcomes between the two groups were independently analyzed to evaluate the associations between clinical outcomes and QTc prolongation.

### Anesthetic, surgical and postoperative management

Standardized anesthesia was performed in accordance with our institutional LT protocol. Standard monitoring devices (peripheral capillary oxygen saturation, 5-lead ECG, non-invasive arterial blood pressure) were applied, and anesthesia was induced with thiopental sodium (5 mg/kg) and maintained with isoflurane titrated to a bispectral index of 40–60. Remifentanil was also infused up to 0.20 mcg/kg/min according to hemodynamic responses. Mechanical ventilation was set at a tidal volume of 8–10 ml/kg using a mixture of medical air and oxygen at a fresh gas flow rate of 2 L/min with the respiratory rate adjusted to maintain normocapnia. The radial artery, femoral artery, femoral vein, and internal jugular vein were cannulated for direct hemodynamic monitoring. Infusions of fluids and vasopressor, such as dopamine, norepinephrine, and vasopressin, were administered to maintain mean arterial pressure ≥ 70 mmHg. A warm blanket and a fluid warmer were used to maintain normothermia with room temperature set at 24°C. Packed red blood cells were transfused when blood hemoglobin concentration was < 8.0 mg/dL. All surgical procedures were in accordance to standardized institutional protocol. Immunosuppression was based on a quadruple regimen: induction with methylprednisolone plus basiliximab and maintenance with tacrolimus starting on postoperative day 3 plus mycophenolate mofetil. The plasma concentration of tacrolimus was titered at 10–15 ng/ml.

### Statistical analysis

Comparisons for continuous variables were made using the t-test or the Wilcoxon rank-sum test, and results were presented as the mean ± standard deviation (SD) or median with interquartile range (IQR). A Chi-square or Fisher’s exact test was used for categorical data. Survival curves were constructed using Kaplan-Meier estimates and compared with the log-rank test. The adjusted hazard ratio (HR) was compared using Cox regression based on the following covariates; male, hypertension, atrial fibrillation, living donor LT, and MELD score which showed *P*-value < 0.05 in the postoperative analysis. Statistical analyses were performed with IBM SPSS 20.0.0 (IBM, Somers, NY) and R version 3.4 (R Foundation) software. All tests were two-tailed and *P* < 0.05 was considered statistically significant.

## Results

**Fig 1:**
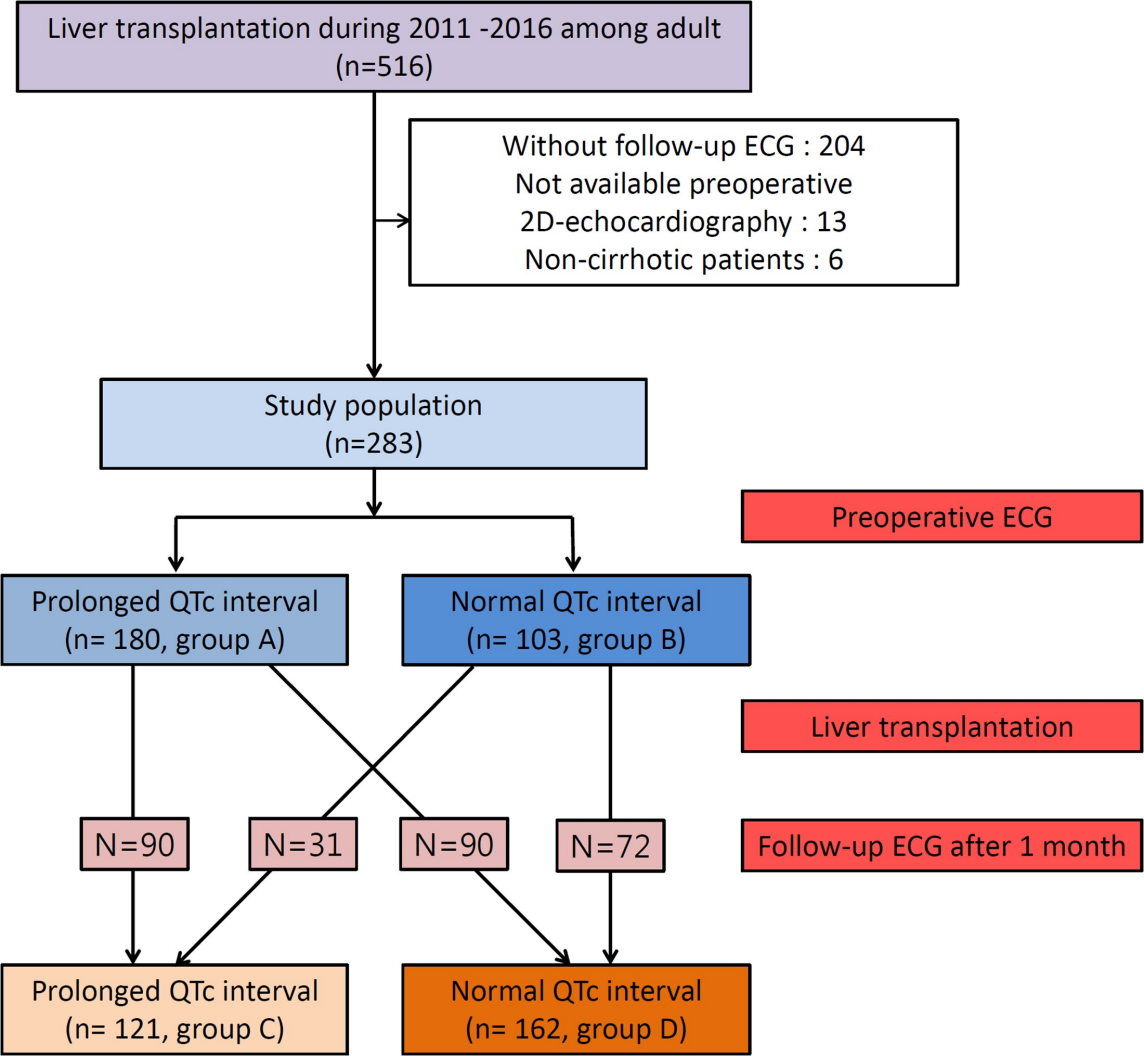
Flow chart of study.

Among 516 consecutive patients, a total of 283 patients were enrolled in the study and the median follow-up was 31 months (IQR 12–52). Fig 1 shows a flow chart of study. We analyzed the times of ECG separately. Among the 283 patients, 180 patients showed a prolonged QTc interval (63.6%, group A), and 103 patients showed a normal QTc interval (36.4%, group B) on preoperative ECG. The mean QTc interval was 467.8ms (±22.73) in the group A and 418.6ms (±17.54) in the group B (*P* < 0.001). On follow-up ECG after 1 month, 121 patients showed a prolonged QTc interval (42.8%, group C), and 162 patients showed a normal QTc interval (57.2%, group D). At follow-up, the mean QTc interval was 464.4ms (±19.53) in the group C and 413.0ms (±18.06) in the group D (*P* < 0.001). In addition, we divided patients into 4 groups according to change in QTc: no change in normal QTc (n = 90, 31.8%), prolonged QTc to normal QTc (n = 90, 31.8%), normal QTc to prolonged QTc (n = 31, 11.0%), and persistent prolonged QTc (n = 72, 25.4%). In these subgroups, we analyzed baseline differences and outcomes.

### Analysis of preoperative ECG: Group A vs. group B

**Fig 2:**
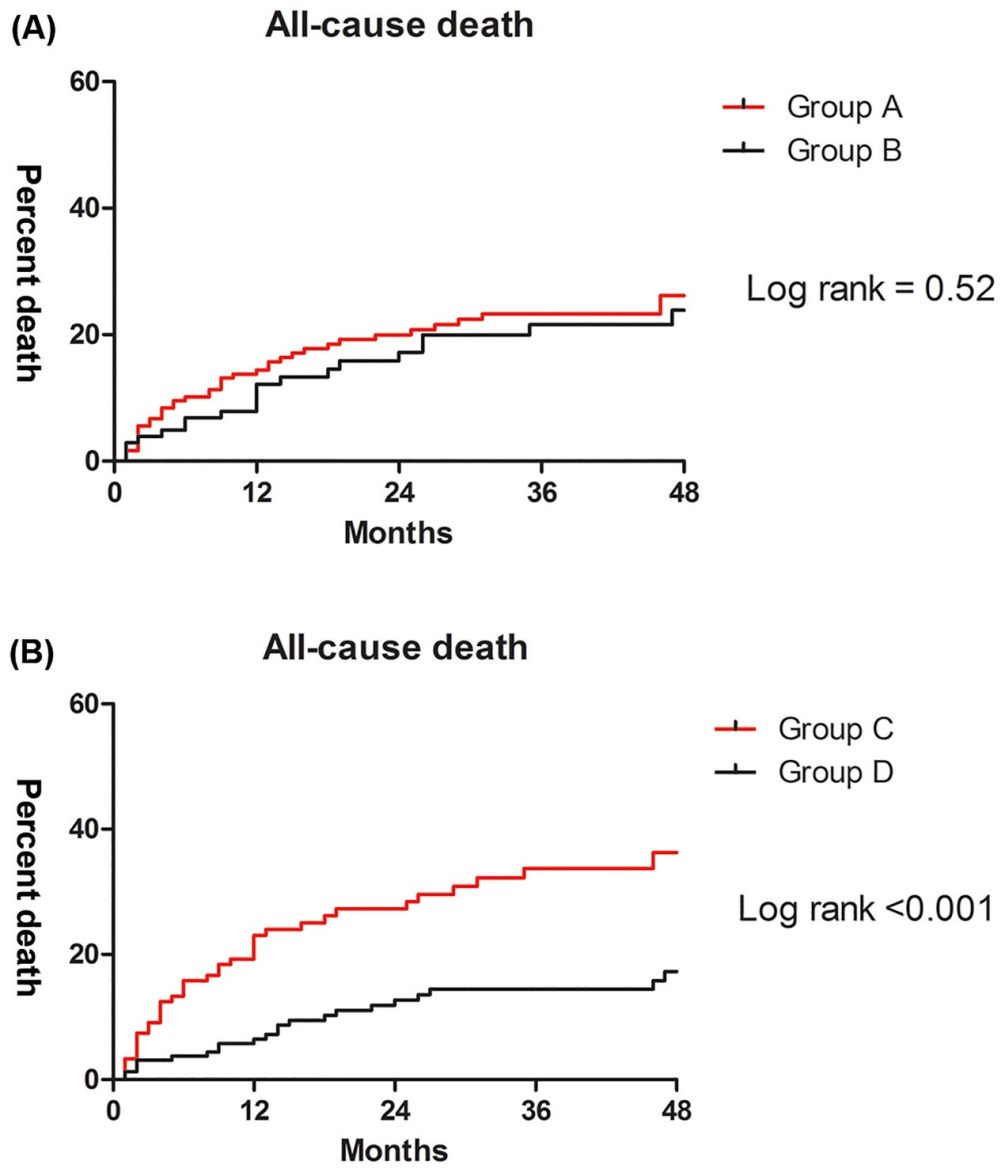
Kaplan-Meier curves for the normal QTc (black) and prolonged QTc groups (red). Curves for the (A) group A and group B, and the (B) group C and group D.

The baseline characteristics are described in Table 1. Patients in the group A showed lower incidences of smoking history, diastolic dysfunction, hepatocellular carcinoma and living donor LT; low levels of hemoglobin, sodium, and albumin; higher rate of diabetes, and, ascites; higher left atrial volume index and MELD score; and were more likely to be female. In the multivariate analysis, there was no significant difference in all-cause mortality (hazard ratio [HR], 1.03; 95% confidence interval [CI], 0.59–1.81; *P* = 0.92) between the group A and group B (Table 2, Fig 2A).

**Table 1:** Baseline characteristics between the group A and group B.

	Group A (N = 180)	Group B (N = 103)	*P*-value
Age	55 (50–61)	55 (50–59)	0.48
Male	128 (71.1)	85 (82.5)	0.03
Hypertension	27 (15.0)	12 (11.7)	0.43
Diabetes	53 (29.4)	18 (17.5)	0.03
Smoking	14 (7.8)	16 (15.5)	0.04
Alcohol	24 (13.3)	8 (7.8)	0.16
Atrial fibrillation	4 (2.2)	1 (1.0)	0.44
Medication			
Beta blocker	42 (23.3)	28 (27.2)	0.47
Calcium channel blocker	7 (3.9)	6 (5.8)	0.45
ACEi/ARB	5 (2.8)	8 (7.8)	0.05
Echocardiography			
Left ventricular ejection fraction < 50	1 (0.6)	1 (1.0)	0.69
Diastolic dysfunction	83 (46.1)	62 (60.2)	0.02
Left atrial volume index (ml/m2)*	38.8 (29.4–47.2)	34.1 (28.7–41.0)	0.01
Valvular heart disease (moderate to severe)	2 (1.1)	0	0.28
Left ventricle enlargement by M-mode	7 (3.9)	4 (3.9)	0.99
Living donor liver transplantation	122 (67.8)	85 (82.5)	0.01
Hepatocellular calcinoma	88 (48.9)	78 (75.7)	<0.001
Ascites	120 (66.7)	40 (38.8)	<0.001
MELD score	18 (13–29)	9 (8–15)	<0.001
Preoperative hemoglobin (g/dL)	10.4 (8.8–11.9)	12.3 (10.0–13.9)	<0.001
Preoperative sodium (mmol/L)	138 (133–141)	141 (137–142)	<0.001
Preoperative pottasium (mmol/L)	3.7 (4.0–4.4)	4.0 (3.8–4.3)	0.72
Albumin (g/dL)	3.1 (2.9–3.5)	3.6 (3.2–3.9)	<0.001

Variables are n(%) or median(interquatile range)

ECG = electrocardiogram; ACEi = angiotensin converting enzyme inhibitor; ARB = angiotensin receptor blocker; MELD = Model for end stage liver disease.

*LAVI was measured in a total of 271 patients.

**Table 2:** Preoperative QTc prolongation and variables associated with clinical outcome.

	Unadjusted HR (95% CI)	*P*-value	Adjusted HR (95% CI)*	*P*-value
All-cause death (n = 64)				
Preoperative QTc prolongation	1.16 (0.69–1.94)	0.58	1.03 (0.59–1.81)	0.92
Atrial fibrillation	0.84 (0.12–6.03)	0.86	0.52 (0.07–3.87)	0.52
Male	0.97 (0.55–1.71)	0.93	1.06 (0.59–1.91)	0.84
Hypertension	2.02 (1.12–3.66)	0.02	2.12 (1.16–3.86)	0.01
MELD score	1.01 (0.99–1.04)	0.19	1.02 (0.99–1.04)	0.24
Living donor liver transplantation	0.85 (0.49–1.46)	0.55	0.98 (0.53–1.84)	0.96

QTc = corrected QT interval; ECG = electrocardiogram; MELD = model for end-stage liver disease; HR = hazard ratio; CI = confidence interval.

*Covariates include male, atrial fibrillation, hypertension, MELD score, and living donor liver transplantation.

### Analysis of follow-up ECGs after LT: Group C vs. group D

Table 3 shows the baseline characteristics between the two groups. Patients in the group C showed lower incidences of atrial fibrillation and living donor LT; lower levels of hemoglobin, sodium, and albumin; higher rates of hypertension; higher MELD scores; and were more likely to be female. In the multivariate analysis, all-cause mortality during the follow-up period was higher in the group C than in the group D (HR, 2.45; 95%CI, 1.43–4.18; *P* = 0.001) (Table 4, Fig 2B).

**Table 3:** Baseline characteristics between the group C and group D.

	Group C (N = 121)	Group D (N = 162)	*P*-value
Age	56 (50–62)	55 (50–59)	0.2
Male	81 (66.9)	132 (81.5)	0.01
Hypertension	24 (19.8)	15 (9.3)	0.01
Diabetes	32 (26.4)	39 (24.1)	0.65
Smoking	11 (9.1)	19 (11.7)	0.48
Alcohol	15 (12.4)	17 (10.5)	0.62
Atrial fibrillation	0	5 (3.1)	0.05
Medication			
Beta blocker	24 (19.8)	46 (28.4)	0.1
Calcium channel blocker	7 (5.8)	6 (3.7)	0.41
ACEi/ARB	4 (3.3)	9 (5.6)	0.37
Echocardiography			
Left ventricular ejection fraction < 50	0	2 (1.2)	0.22
Diastolic dysfunction	57 (47.1)	88 (54.3)	0.23
Left atrial volume index (ml/m2)*	37.0 (28.8–46.6)	36.0 (29.2–44.3)	0.51
Valvular heart disease (moderate to severe)	1 (0.8)	1 (0.6)	0.84
Left ventricle enlargement by M-mode	5 (4.1)	6 (3.7)	0.85
Living donor liver transplantation	78 (64.5)	129 (79.6)	0.004
Hepatocellular calcinoma	64 (52.9)	102 (63.0)	0.09
Ascites	72 (59.5)	88 (54.3)	0.38
MELD score	17 (11–30)	14 (9–20)	0.002
Hemoglobin on follow up (g/dL)	9.1 (8.3–10.7)	10.2 (8.7–11.9)	0.01
Sodium on follow up(mmol/L)	136 (134–139)	137 (134–139)	0.03
Pottasium on follow up (mmol/L)	4.5 (4.2–5.0)	4.6 (4.2–5.0)	0.09
Albumin on follow-up (g/dL)	3.2 (2.9–3.5)	3.3 (3.0–3.7)	0.01

Variables are n(%) or median(interquatile range)

ACEi = angiotensin converting enzyme inhibitor; ARB = angiotensin receptor blocker; Model for end stage liver disease.

*LAVI was measured in a total of 271 patients.

**Table 4:** QTc prolongation on follow-up ECG and variables associated with clinical outcome.

	Unadjusted HR (95% CI)	*P*-value	Adjusted HR (95% CI)*	*P*-value
All-cause death (n = 64)				
QTc prolongation on follow-up ECG	2.60 (1.57–4.32)	<0.001	2.45 (1.43–4.18)	0.001
Atrial fibrillation	0.84 (0.12–6.03)	0.86	0.99 (0.13–7.70)	0.99
Male	0.97 (0.55–1.71)	0.93	1.19 (0.66–2.13)	0.56
Hypertension	2.02 (1.12–3.66)	0.02	1.75 (0.96–3.22)	0.07
MELD score	1.01 (0.99–1.04)	0.19	1.01 (0.99–1.03)	0.39
Living donor liver transplantation	0.85 (0.49–1.46)	0.55	1.05 (0.56–1.97)	0.88

QTc = corrected QT interval; ECG = electrocardiogram; MELD = model for end-stage liver disease; HR = hazard ratio; CI = confidence interval.

*Covariates include male, atrial fibrillation, hypertension, MELD score, and living donor liver transplantation.

### Analysis of changes in QTc interval

**Fig 3:**
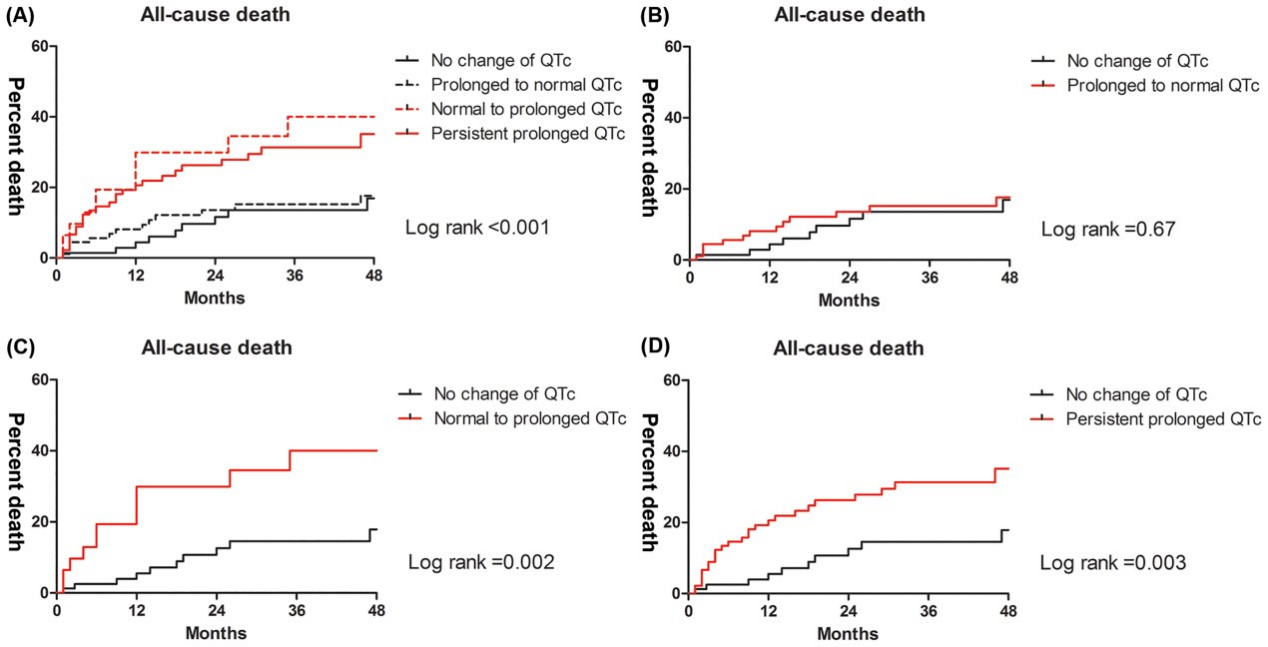
Kaplan-Meier curves according to change in QTc interval before and after liver transplantation. Curves for (A) all four groups, (B) no change and normal to prolonged, (C) no change and prolonged to normal, and (D) no change and persistent prolonged.

Among the patient group that had a prolonged QTc preoperatively, a comparison between patients with normal and prolonged QTc intervals on follow-up ECG are shown in S1 Table. The prolonged QTc group was more likely female and showed lower incidences of atrial fibrillation and of low preoperative hemoglobin levels. A calcium channel blocker was more frequently used in the prolonged QTc group than in the normal QTc group. Among the patient group with normal preoperative QTc intervals, a comparison between patients with normal and prolonged QTc intervals on follow-up ECG is shown in S2 Table. Lower serum potassium levels were seen in the prolonged QTc group. Fig 3 shows the Kaplan-Meier curves between the four groups. Patients who had a prolonged QTc interval on follow-up ECG showed increased mortality regardless of whether preoperative QTc prolongation was observed. Table 5 shows causes of death between the four groups. Among the patients who had a prolonged QTc interval after LT (either the normal to prolonged QTc group or the persistent, prolonged QTc group), over 40% died due to sepsis. Cardiac deaths occurred only in patients with a prolonged QTc interval on follow-up ECG.

**Table 5:** Causes of death between 4 groups according to change of QTc.

	No change of normal QTc (N = 72)	Normal to prolonged QTc (N = 31)	Prolonged to normal QTc (N = 90)	Persistent prolonged QTc (N = 90)
All cause-death	10 (13.9)	12 (38.7)	14 (15.6)	28 (31.1)
Cause of death				
Sepsis	2 (20.0)	5 (41.7)	4 (28.6)	12 (42.9)
Hepatocellular carcinoma	4 (40.0)	3 (25.0)	4 (28.6)	6 (21.5)
Bleeding	2 (20.0)	0	0	2 (7.1)
Rejection or hepatic failure	1 (10.0)	3 (25.0)	4 (28.6)	3 (10.7)
Cardiac death	0	1 (8.3)	0	3 (10.7)
Others	1 (10.0)	0	2 (14.2)	2 (7.1)

Variables are n(%)

## Discussion

The main findings of this study were as follows: 1) preoperative QTc prolongation was not associated with mortality in LC patients who underwent LT; 2) patients who had QTc prolongation on the LT postoperative follow-up ECG showed an increased incidence of all-cause death; and 3) regardless of preoperative QTc prolongation, QTc prolongation after LT was independently associated with all-cause mortality in patients with LC.

In a healthy population, prolonged QTc is associated with all-cause and cardiovascular mortality [4,15]. In addition, prolonged QTc interval may be an important marker of cerebral damage [16]. In LC patients, prolonged QTc interval is more frequently seen as a result of several features of LC, such as portal hypertension, sympathetic nervous system activation, or autonomic neuropathy attributed to ventricular repolarization [6,17,18]. Several clinical trials have shown that there is an association of prolonged QTc interval and all-cause or cardiovascular mortality in patients with LC [3,6]. However, the association between QTc prolongation and mortality was not consistent in patients receiving LT [10,19,20]. Moreover, there is limited data on the significance of QTc prolongation in follow-up ECG after LT. A previous study had shown that an improvement in QTc interval post-transplant did not translate into a mortality benefit. However, the study was a case-control study with a small patient population of 73 [20].

As mentioned above, a prolonged QTc interval on preoperative ECG was not associated all-cause mortality in LC patients who underwent LT [10,19,20]. We showed a similar result here. QTc prolongation is a supportive criterion of cirrhotic cardiomyopathy, and Child-Pugh score and plasma norepinephrine concentration, a marker of sympathetic nervous system activity, were independently associated with the abnormality [3]. LT has been shown to eventually reverse cardiac dysfunction and normalize the QT interval in some patients [1]. Hence, it is reasonable that preoperative QTc prolongation showed no correlation with mortality after LT. However, there is uncertainty about which patients showed a normalized QTc interval after LT and whether a prolonged QTc interval after LT is associated with a poor clinical outcome.

In some LC patients, prolonged QTc interval could be normalized after successful LT [1,10]. In the present study, 50% of patients showed normalization of QTc interval after LT. We showed that prolonged QTc interval on follow-up ECG was associated with a poor clinical outcome in patients who underwent LT. There are several possible explanations for this result. First, individuals with prolonged ventricular repolarization on surface ECG by prolongation of the QT interval are predisposed to ventricular fibrillation and sudden death [4]. Second, a prolonged QT interval is associated not only with sudden death but also with all-cause mortality, which was shown recently in a retrospective study [21]. The main causes of mortality were cerebral stroke/head trauma and heart failure in addition to an aborted cardiac arrest in patients with QT prolongation. Hence, we expect that QT prolongation is associated with mortality in patients with severe illness, although we do not know exact mechanism. Electrolyte imbalance or fatal arrhythmia in conjuntion with severe illness may contribute to increased mortality, but further investigation is needed. Furthermore, prolonged QTc interval is associated with a poor clinical outcome in patients with sepsis [22]. In the present study, over 40% of patients who had prolonged QTc interval on follow-up ECG died from sepsis. Several antibiotics that are used in septic patients could aggravate prolonged QT interval and contribute to death [3,23]. Finally, QT prolongation showed association with severity of LC. [24] Even in the early stage of LC, QT prolongation may be present. Therefore, prolonged QT interval may reflect liver damage itself and lead to poor clinical outcome in the patients with LC even in LT patients. In consideration of our results, clinicians need to be aware of the effect of QTc prolongation and proceed cautiously with LT patients. The definition of QTc interval prolongation varies [16,22,23].

The following limitations apply to this study. First, this study was not randomized; therefore, potential confounding factors or selection bias might have significantly affected the results. However, it is hard to match all the clinical variables, especially for patients under critical conditions like severe LC, hepatic failure, or post-operative status. Furthermore, our study population might be homogenous in some way considering that all surgical procedures and post-surgical management were in accordance to standardized institutional protocol. Second, a QTc interval calculated by the Bazett formula is highly dependent on heart rate. Hence, critical situations that affect heart rate may have confounded the results. Third, we cannot exclude that potential pre-transplant underdiagnosis and/or undertreatment of ischemic heart disease or cardiac arrhythmia may have led to an increased post-transplant incidence of mortality. Fourth, the differences in the incidence of cardiac death were not analyzed since only a few cardiac deaths were observed. Fifth, inherent limitations are associated with 12-lead ECG measurements of QT intervals, and rate-correction of QT intervals are often performed incorrectly [25]. Sixth, not only a follow-up ECG after LT was not available in all patients but also not all follow-up ECGs were evaluated at same timepoint after LT. Lastly, the present study did not determine whether the management of QTc prolongation could benefit mortality rates. Despite these limitations, the present study suggests that postoperative QTc prolongation is a useful surrogate marker of mortality, and that it can inform the current prognostic evaluation of LC patients undergoing LT.

## Conclusion

In patients who underwent LT for LC, postoperative QTc prolongation on ECG, rather than preoperative, is associated with mortality. Larger registry datasets are needed to support this finding.

## Supporting information

S1 Table Comparison between normalized QTc and prolonged QTc after liver transplantation among abnormal QTc before operation. (DOCX)

S2 Table Comparison between normal QTc and prolonged QTc after liver transplantation among normal QTc before operation. (DOCX)

## Abbreviations

CI: confidence interval
ECG: electrocardiogram
HR: hazard ratio
IQR: interquartile range
LC: liver cirrhosis
LT: liver transplantation
MELD: model for end-stage liver disease
QTc: corrected QT for heart rate
SD: standard deviation
