# Supplementary material for: Corrected QT interval on the electrocardiogram after liver transplantation: Surrogate marker of poor clinical outcomes?
Source: PLoS One. 2018 Oct 26;13(10):e0206463. doi: 10.1371/journal.pone.0206463 (PMC6203397; doi:10.1371/journal.pone.0206463)
Supplement: S1 Table — (DOCX) [file pone.0206463.s001.docx]

**S1 Table. Comparison between normalized QTc and prolonged QTc after liver transplantation among abnormal QTc before operation.**

|  | **Normalized QTc (N = 90)** | **Prolonged QTc (N = 90)** | ***P* value** |
| --- | --- | --- | --- |
| Age | 54 (50-60) | 56 (51-62) | 0.26 |
| Male | 71 (78.9) | 57 (63.3) | 0.02 |
| Hypertension | 6 (6.7) | 21 (23.3) | 0.002 |
| Diabetes | 25 (27.8) | 28 (31.1) | 0.62 |
| Smoking | 9 (10.0) | 5 (5.6) | 0.27 |
| Alcohol | 12 (13.3) | 12 (13.3) | 0.99 |
| Atrial fibrillation | 4 (4.4) | 0 | 0.04 |
| Heart rate on ECG | 69 (62-84) | 76 (66-88) | 0.06 |
| Medication |  |  |  |
| Beta blocker | 26 (28.9) | 16 (17.8) | 0.08 |
| Calcium channel blocker | 1 (1.1) | 6 (6.7) | 0.05 |
| ACEi/ARB | 3 (3.3) | 2 (2.2) | 0.65 |
| Echocardiography |  |  |  |
| Left ventricular ejection fraction < 50 | 1 (1.1) | 0 | 0.32 |
| Diastolic dysfunction | 44 (48.9) | 39 (43.3) | 0.46 |
| Left atrial volume index (ml/m2)* | 39.3 (30.0-46.5) | 38.5 (29.3-48.4) | 0.83 |
| Valvular heart disease (moderate to severe) | 1 (1.1) | 1 (1.1) | 0.99 |
| Left ventricle enlargement by M-mode | 3 (3.4) | 4 (4.4) | 0.71 |
| Living donor liver transplantation | 67 (74.4) | 55 (61.1) | 0.06 |
| Hepatocellular calcinoma | 46 (51.1) | 42 (46.7) | 0.55 |
| Ascites | 62 (68.9) | 58 (64.4) | 0.53 |
| MELD score | 17 (13-24) | 20 (14-32) | 0.1 |
| Hemoglobin on follow up (q/dL) | 10.5 (9.0-11.7) | 9.8 (8.3-11.1) | 0.01 |
| Sodium on follow up(mmol/L) | 136 (134-139) | 136 (134-139) | 0.26 |
| Pottasium on follow up (mmol/L) | 4.6 (4.2-5.1) | 4.6 (4.3-5.1) | 0.99 |
| Albumin on follow-up (q/dL) | 3.2 (2.9-3.6) | 3.2 (2.9-3.5) | 0.51 |

Variables are n(%) or median(interquatile range)

ACEi = angiotensin converting enzyme inhibitor; ARB = angiotensin receptor blocker; Model for end stage liver disease.
