# Supplementary material for: Corrected QT interval on the electrocardiogram after liver transplantation: Surrogate marker of poor clinical outcomes?
Source: PLoS One. 2018 Oct 26;13(10):e0206463. doi: 10.1371/journal.pone.0206463 (PMC6203397; doi:10.1371/journal.pone.0206463)
Supplement: S2 Table — (DOCX) [file pone.0206463.s002.docx]

**S2 Table. Comparison between normal QTc and prolonged QTc after liver transplantation among normal QTc before operation.**

|  | **Normal QTc (N = 72)** | **Prolonged QTc (N = 31)** | ***P*-value** |
| --- | --- | --- | --- |
| Age | 55 (50-58) | 55 (48-62) | 0.69 |
| Male | 61 (84.7) | 24 (77.4) | 0.37 |
| Hypertension | 9 (12.5) | 3 (9.7) | 0.68 |
| Diabetes | 14 (19.4) | 4 (12.9) | 0.42 |
| Smoking | 10 (13.9) | 6 (19.4) | 0.48 |
| Alcohol | 5 (6.9) | 3 (9.7) | 0.64 |
| Atrial fibrillation | 1 (1.4) | 0 | 0.51 |
| Heart rate on ECG | 60 (55-70) | 64 (58-74) | 0.1 |
| Medication |  |  |  |
| Beta blocker | 20 (27.8) | 8 (25.8) | 0.84 |
| Calcium channel blocker | 5 (6.9) | 1 (3.2) | 0.46 |
| ACEi/ARB | 6 (8.3) | 2 (6.5) | 0.74 |
| Echocardiography |  |  |  |
| Left ventricular ejection fraction < 50 | 1 (1.4) | 0 | 0.51 |
| Diastolic dysfunction | 44 (61.1) | 18 (58.1) | 0.77 |
| Left atrial volume index (ml/m2)* | 34.0 (28.6-41.0) | 34.7 (28.5-41.7) | 0.95 |
| Valvular heart disease (moderate to severe) | 0 | 0 |  |
| Left ventricle enlargement by M-mode | 3 (4.2) | 1 (3.3) | 0.84 |
| Living donor liver transplantation | 62 (86.1) | 23 (74.2) | 0.14 |
| Hepatocellular calcinoma | 56 (77.8) | 22 (71.0) | 0.46 |
| Ascites | 26 (36.1) | 14 (45.2) | 0.39 |
| MELD score | 9 (7-15) | 10 (8-16) | 0.11 |
| Hemoglobin on follow up (q/dL) | 11.2 (10.0-12.4) | 10.8 (9.9-11.8) | 0.26 |
| Sodium on follow up(mmol/L) | 138 (135-140) | 138 (134-140) | 0.7 |
| Pottasium on follow up (mmol/L) | 4.5 (4.3-4.8) | 4.3 (4.0-4.7) | 0.01 |
| Albumin on follow-up (q/dL) | 3.4 (3.1-3.8) | 3.4 (2.8-3.5) | 0.16 |

Variables are n(%) or median(interquatile range)

ACEi = angiotensin converting enzyme inhibitor; ARB = angiotensin receptor blocker; Model for end stage liver disease
